# Mutations in the Control of Virulence Sensor Gene from *Streptococcus pyogenes* after Infection in Mice Lead to Clonal Bacterial Variants with Altered Gene Regulatory Activity and Virulence

**DOI:** 10.1371/journal.pone.0100698

**Published:** 2014-06-26

**Authors:** Jeffrey A. Mayfield, Zhong Liang, Garima Agrahari, Shaun W. Lee, Deborah L. Donahue, Victoria A. Ploplis, Francis J. Castellino

**Affiliations:** 1 W. M. Keck Center for Transgene Research, University of Notre Dame, Notre Dame, Indiana, United States of America; 2 Department of Chemistry and Biochemistry, University of Notre Dame, Notre Dame, Indiana, United States of America; 3 Department of Biological Sciences, University of Notre Dame, Notre Dame, Indiana, United States of America; Columbia University, United States of America

## Abstract

The cluster of virulence sensor (CovS)/responder (CovR) two-component operon (CovRS) regulates ∼15% of the genes of the Group A *Streptococcal pyogenes* (GAS) genome. Bacterial clones containing inactivating mutations in the *covS* gene have been isolated from patients with virulent invasive diseases. We report herein an assessment of the nature and types of *covS* mutations that can occur in both virulent and nonvirulent GAS strains, and assess whether a nonvirulent GAS can attain enhanced virulence through this mechanism. A group of mice were infected with a globally-disseminated clonal M1T1 GAS (isolate 5448), containing wild-type (WT) CovRS (5448/CovR^+^S^+^), or less virulent engineered GAS strains, AP53/CovR^+^S^+^ and Manfredo M5/CovR^+^S^+^. SpeB negative GAS clones from wound sites and/or from bacteria disseminated to the spleen were isolated and the *covS* gene was subjected to DNA sequence analysis. Numerous examples of inactivating mutations were found in CovS in all regions of the gene. The mutations found included frame-shift insertions and deletions, and in-frame small and large deletions in the gene. Many of the mutations found resulted in early translation termination of CovS. Thus, the *covS* gene is a genomic mutagenic target that gives GAS enhanced virulence. In cases wherein CovS^−^ was discovered, these clonal variants exhibited high lethality, further suggesting that randomly mutated *covS* genes occur during the course of infection, and lead to the development of a more invasive infection.

## Introduction

Two-component signal-transducing systems (TCS) are found throughout the bacterial world and regulate a number of critical virulence processes, thereby permitting these organisms to survive under the harsh conditions inflicted by the host in its defense. In general, TCS are composed of an extracellular membrane-bound sensor protein and an intracellular nuclear regulatory responder protein [1]. The control of virulence TCS operon (*covRS*) found in the strict human pathogen, *Streptococcus pyogenes* (Group A *Streptococcus*; GAS), is critical for the regulation of ∼15% of the GAS genome [2]. Mutations in CovRS that confer phenotypic heterogeneity in the invading bacterial population can assist the bacteria in evading the defense responses of the host, thereby allowing the microorganism to disseminate into deep tissue. Critical GAS virulence genes, *e.g.*, streptokinase (*ska*), streptococcal pyrogenic exotoxin (*spe*)B, and hyaluronic acid capsule biosynthesis genes (*hasABC*), as well as some GAS-adopted prophage genes, *e.g.*, streptodornase (*sda1*), have been shown to be under the control of CovRS [2]. GAS is the causative agent for a number of illnesses, ranging from minor treatable throat and skin infections to more severe diseases, such as streptococcal toxic shock syndrome and necrotizing *fasciitis*. These maladies are growing concerns due to establishment of clonal hypervirulent GAS strains. A better understanding of mechanisms of gene regulation employed by GAS, especially by TCS, such as CovRS, that can serve to rapidly alter bacterial gene expression under host defense pressures, will enable novel approaches to better combat this serious threat to human health.

In contrast to most regulatory systems, CovR, a cytoplasmic transcriptional regulator, represses most of its gene targets. This repression is attenuated when the bacteria are under duress in the host, causing an increase in expression of virulence gene products. The sensor component (CovS) of CovRS is proposed to recognize stress signals, *e.g.*, elevated [Mg^2+^], temperature, low tissue O_2_ tension in tissue, and salinity, upon which CovS autophosphorylates and then transfers this phosphoryl group to CovR, thus altering the affinity of CovR for promoters of its gene targets [3], [4]. The complexities in environmental stimuli and specific gene regulation in the CovRS system are not well understood, and remain a topic of some debate [5]. It has been shown that when experiencing environmental pressures during host infection, GAS clones are selected towards those that lack a fully functional CovS component [6]. Several hypervirulent GAS clinical and animal-passaged clones have been isolated with mutations in the *covS* gene that attenuate the activity of its gene product toward CovR [7]–[11]. One gene under CovRS transcriptional regulation is *speB*, which encodes an extracellular cysteine protease (SpeB) that catalyzes cleavage of a number of host and GAS proteins [12], [13]. Several roles for SpeB have been observed in establishing infection, including a role in evading the host autophagy pathway [14] and likely many more will be determined; however, it has been documented that hypervirulent GAS strains with inactivated CovS (CovR^+^S^−^) lack detectable SpeB in culture supernatants [9]. Intact CovR^+^S^+^ greatly enhances *speB* expression, which is necessary for establishing early infectivity, after which downregulation of SpeB expression promotes virulence. This switch in *speB* expression is facilitated by *covS* gene mutations, which then allows CovR to repress *speB* expression and thereby permit other virulence factors, *e.g.*, the products of the *sda1*, streptokinase (*ska*), and streptolysin-O (*slo*) genes, to avoid cleavage by SpeB, thus sustaining their activities as GAS virulence factors. This selection for *covS* mutants suggests that only colonies that are maximally discharging their virulence factors are able to survive in host compartments that are particularly hostile to the microorganism. However, under conditions where host immune surveillance is not present, this sensor protein is likely important for alleviating the energy burden of producing virulence gene products and allows for long-term colonization.

A number of hypervirulent GAS isolates have been characterized by inactivating mutations in CovR or CovS, and it is unclear whether these mutations follow any consistent patterns. The goal of this study was to employ single well-characterized strains of GAS to extensively assess whether consistencies of gene mutations occur in *covS* during host infection that result in hypervirulent strains. Using the invasive globally-disseminated M1T1 GAS strain, *viz.*, 5448/CovR^+^S^+^, and the nonvirulent engineered strain, AP53/CovR^+^S^+^, both of which contain full-length and fully-functional *covS* genes, we performed a number of mouse-passage (MP) studies where the same wild-type (WT) bacteria were used to infect different mice. Infection and dissemination sites were examined for SpeB-negative (SpeB^−^) clones. These clones were then further characterized to determine the nature of gene mutations in *covS*. A number of such isolates were recovered, further confirmed by increased *sda1* and *ska* expression and activity. The results suggest that many types of mutations can occur in the *covS* gene, leading to the hypothesis that, when establishing infection, the entire *covS* gene is susceptible to mutations, and clones containing CovS-inactivating mutations have the ability to affect numerous end-points that lead to serious invasive disease and resist innate defenses.

## Materials and Methods

### Bacterial strains and culture

GAS serotype M1T1, strain 5448/*covR^+^S^+^/emm1* ^+^, has been previously isolated from a patient with an invasive GAS infection [15], [16]. This GAS strain also contains the lysogenically-inserted prophage-encoded genes, *speA* and *sdaI*, which are independent virulence factors. GAS strain Manfredo/*covR^+^S^+^/emm5*, from an GAS avirulent infection [17], was provided by Dr. G. Lindahl (Lund, SW). The clinical GAS isolates, MGAS315/*emm3* ^+^ [18], JRS4/*emm6^+^* [19], and MGAS8232/*emm18^+^* [20], were provided by Dr. J.R. Scott (Emory University). AP53/*covR^+^S^−^/emm53(pam)^+^* has been previously described as a primary isolate from a patient with severe invasive disease. As obtained, we found that this latter isolate has an inactivating mutation in the *covS* gene (ΔT^1404^), leading to a premature end of translation at amino acid-469 in the CovS protein, and is highly virulent, despite its lack of the particular prophage insertions that provide *sda1* and *speA*. This *covS* ^−^ gene was recomplemented to nonvirulent AP53/*covR^+^S^+^/emm53(pam)^+^*, through bacterial mutagenesis, in which we reinserted T^1404^ in *covS^−^* and restored full CovR^+^S^+^ function to GAS AP53 [21].

All strains were routinely grown on 5% sheep blood agar (SBA) plates (Teknova, Hollister, CA) and cultured at 37°C and 5% CO_2_ in Todd-Hewitt broth (BD Biosciences, San Jose, CA), supplemented with 10% yeast (THY). To screen for SpeB proteolytic activity, GAS isolates were plated on THY/milk (3% skim milk) agar plates for ∼24 hr at 37°C and 5% CO_2_. The strains used in this study are summarized in Table 1.

**Table 1:** *Streptococcus pyogenes* strains used in this study.

Strain Name	*emm* Type	Reference
5448/*covR^+^S^+^*	1	14
Manfredo/*covR^+^S^+^*	5	16
MGAS315	3	17
JRS4	6	18
MGAS8232	18	19
AP53/*covR^+^S^−^*	53	20
AP53/*covR^+^S^+^*	53	20
AP53/*covR^+^S^+^*/*(pam)^−^*	53	20

### Subcutaneous infection and organ processing

Overnight cultures of GAS 5448/CovR^+^S^+^, grown from a single colony, were used to inoculate (1∶10) 30 ml of pre-warmed THY media. The cells were grown to mid-log phase (LP; A_600nm_ ∼0.6), pelleted by centrifugation at 2,500×g for 10 min, then washed 2× in sterile 0.7% saline, followed by resuspension in a final volume sufficient to provide ∼1–2×10^9^ CFU/ml, as later determined by plating on SBA and counting colonies from serial dilutions. Cohorts of C57Bl/6 mice containing the human plasminogen (hPg) transgene [C57Bl/6(hPg(Tg)] [22], provided by Prof. D. Ginsburg (Ann Arbor, MI), were subcutaneously injected with a dose of ∼1–2×10^8^ CFU/100 µl. At a time of 3 days post-infection, mice were sacrificed, and the skin wounds, livers, and spleens were harvested and placed into 2 ml of 0.7% saline. Samples were dispersed using a homogenizer (IKA, Wilmington, NC) for ∼30 sec at 24,000 rpm on ice. Homogenates were serially diluted and plated for colony counting. Significance of dissemination count differences between organs was determined using an unpaired two-tailed student's t-test. In addition to SBA plating, dilutions were plated on THY milk/agar to detect SpeB proteolytic activity.

### SpeB activity assays

Mouse-passaged GAS colonies were visually screened to tentatively identify SpeB^−^ clones by the lack of a visual lytic (clearing) zone in THY milk/agar after a 24 hr incubation at 37°C in a 5% CO_2_ atmosphere. To confirm the presence or absence of SpeB, lytic-negative colonies were cultured in 10 ml of THY media overnight. Following centrifugation at 2,500×g for 10 min, bacterial supernatants were sterile-filtered and concentrated (∼10-fold) using centrifugal concentrator devices (Millipore, Billerica, MA) with a 10K molecular weight cutoff. Concentrated supernatants were incubated in activation buffer (1 mM EDTA, 10 mM DTT, 0.1 M acetate, pH 5) for 30 min at 37°C prior to addition of 0.6 mM of the chromogenic substrate, N-benzoyl-proline-phenylalanine-arginine-*p*-nitroanilide-HCl (Sigma, St. Louis, MO). Amidolytic activity was continuously monitored at A_405nm_. This was repeated at least 2× for each sample with the addition of 28 µM cysteine protease inhibitor, E64, (Sigma-Aldrich, St. Louis, MO) to confirm specificity for SpeB.

### *covS* gene sequencing

Genomic DNA (gDNA) was obtained *via* freeze-thaw cell lysis of SpeB^−^ bacterial isolates. Primers specific for the *covRS* region (876F-GTGCGTGGCATGGGATACGT and 2451R-CCTGTCACATTAACAATGCCTT) were used to amplify the gene product in all strains by PCR. To identify mutations, nucleotide sequences were obtained using the same primers for all GAS strains. PCR and subsequent sequencing was performed in duplicate or triplicate to confirm the positions and identities of the mutations. Two primers were used to determine the presence or absence of *covS* mutations in inoculum prior to infection (84F-CTGCATTTTCTCTGCCTTTACACTG and 1195R-GTCAATCAGAATCATCAAAGCCTGC). These reactions were run using gDNA isolated from overnight cultures of multiple GAS colonies.

### DNase activity assays

Sda1-catalyzed DNase activities of the GAS 5448 *covS* mutants were performed as previously described [23]. Strains 5448/CovR^+^S^+^ and MP mutants of this strain were grown to LP (A_600nm_ ∼0.6) and centrifuged at 2,500×g, after which the supernatants were removed and sterile-filtered. Approximately 1 µl of supernatant was incubated with 1 µg of calf thymus DNA for 5 min at 37°C in 300 mM Tris-Cl/3 mM MgCl_2_/3 mM CaCl_2_, pH 8.0. Enzymatic reactions were terminated by addition of 100 mM EDTA. The samples were then subjected to electrophoresis on 1.5% agarose gels and bands were detected *via* ethidium bromide stain.

### Polymerase Chain Reaction (PCR) and Reverse Transcription-PCR (RT-PCR)

Total RNA was isolated as described [21] from two independent cultures of WT 5448/*covR^+^S* ^+^ and two of its mouse-passaged *covR^+^S^−^* mutants, *viz.*, #12 (skin) and #14 (skin), grown to A_600nm_ ∼0.6. The sample was treated twice with DNase to eliminate any contaminating gDNA.

Total genomic DNA (gDNA) was obtained from single GAS colonies of strains that were picked from streaks on sheep blood agar and grown in THY overnight at 37°C. The gDNA was isolated after treatment of the cells with lysozyme/proteinase K and cell lysis buffer (100 mM Tris/5 mM EDTA/0.2% SDS/200 mM NaCl, pH 8.5), and extracted with phenol/chloroform/isoamyl alcohol (25/24/1, v/v/v). The gDNA was precipitated with isopropanol and washed with 70% ethanol.

Primers specific for *sda1* (438F: 5′-GAGAGCCACTGAATCCGACTAC and 688R: 5′-TACTGCATCCCACCTTTAC-GAT, amplicon size, 251 bp), *ska* (55F: 5′-GGAACAGTGAAGCCTGTCCAAGCT and 369R: 5′-GTAGCCGTCGTTACTGTG-AACGTT, amplicon size, 315 bp), and *slo* (2334F: 5′-GCTAGTACAGAAACCAC-AACGAC and 2742R: 5′-GATAGGTCC-TATCAGTGACAGAGTC, amplicon size, 409 bp), along with the reference gene, *gapdh* (*plr*) [21], were used for both the RT-PCR of the cDNA and PCR of the gDNA, which was performed as previously described [21].

### Plasminogen activation assays

To measure the activity of streptokinase (SK) in conditioned GAS supernatants, hPg activation assays were performed as previously published [24]. Briefly, 0.2 µM Glu^1^-hPg, isolated by affinity chromatography [25], was mixed in 96-well plates with 0.25 mM S2251 (*H*-D-Val-Leu-Lys-pNA; Chromogenix, Milan, Italy) in 10 mM Hepes/150 mM NaCl, pH 7.4, at 37°C. The reactions were initiated by addition of 20 µl of supernatants, taken from 30 ml cultures grown to A_600nm_ ∼0.6 and A_600nm_ ∼1.2 in THY broth that had been concentrated (∼10-fold) and resuspended in buffer to remove media components. Human fibrinogen (ERL, South Bend, IN) was added at 1.0 µM to increase activity, since the SK2a produced by this M1 GAS line is stimulated by fibrinogen [24]. After initiation of the activation by conditioned cell supernatants, the reaction was monitored at 405 nm in 1 min intervals for 2 hr at 37°C. The initial velocity (V_i_; mAbs_405nm_min-^2^) was obtained by taking the slope of the linear portion of the activation curve after squaring the time values.

### Mouse survival studies

C57Bl/6(hPg(Tg) male mice (6–10 weeks of age) were injected subcutaneously with ∼10^7^–10^8^ GAS cells. The infected mice were observed for up to 10 days to monitor the occurrence of death. For statistical analysis, Kaplan-Meier survival curves were analyzed by the paired log-rank test using GraphPad PRISM 6 software with p values <0.05 considered significant. These protocols were approved by the Institutional Animal Care and Use Committee (IACUC) of the University of Notre Dame.

Death as an endpoint is used in order to determine survival curves with different strains of bacteria. The survival group was monitored two or more times/day for up to 10 days, after that time any full-term survivors were humanely euthanized as per the procedures established by our local IACUC, *i.e.*, mice were euthanized by a automated CO_2_ system. The criterion used in this study for early euthanasia was placing a mouse on its side and if unable to right itself, or stand on all four legs, the mouse was euthanized by cervical dislocation. Otherwise, mice in this group must progress to death to mimic terminal disease states in patients. Mice continue to eat, drink, and remain in motion even as the disease progresses.

No analgesics were employed in this work. Inflammation is a key experimental component, and any compounds that might alter the inflammatory process would be counterproductive. Great care was taken to use agents that would not interfere with the experimental outcome. A variety of analgesics were considered and it was concluded that: 1) non-steroidal anti-inflammatory agents or opiods cannot be used due to interference with the inflammatory process [26]; 2) butorphanol cannot be used due to seeming interference with inflammation [27]; and 3) buprenorphine cannot be used because it interferes with the inflammatory process [28].

The mice in these experiments were housed with enrichment, such as extra bedding materials. Some feed was placed on the bottom of the cages to enable mice to eat without standing (in case the hind limb is sore from the wound). The water bottle in the cages was very low and mice only needed to extend their neck to drink. Mice were not housed individually since this would induce stress in the mice.

## Results

### Bacterial dissemination readily occurs with GAS 5448/CovR^+^S^+^

**Figure 1:**
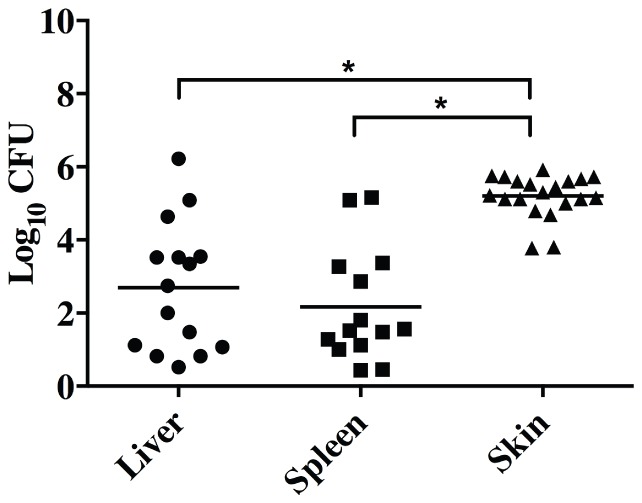
GAS strain 5448 dissemination from skin to deep tissue. Analysis of bacterial loads in liver, spleen, and skin wound tissue. C57Bl/6[hPg(Tg)] mice were injected with a dose of ∼1–2×10^8^ CFU/100 µl of 5448 WT-5448/CovR^+^S^+^. At 3 days post-infection, organs were harvested and bacterial counts obtained from serial dilutions of homogenized tissues. Asterisks indicate statistical significance determined by unpaired two-tailed students t-test, P<0.001.

Using the highly virulent GAS, 5448/CovR^+^S^+^, to infect mice, bacterial dissemination from skin to deep tissue occurs within a three day period, yielding measureable levels of GAS colony forming units (CFU) in liver and spleen tissue. Representative plots of the bacterial counts taken from all tissues are shown in Figure 1, with each point representing a different mouse. On average, significantly more bacteria were recovered from the skin lesion (∼2×10^5^ CFU) than from the deeper tissues, *viz.*, liver and spleen, which yielded ∼9×10^4^ and ∼1×10^4^ CFU, respectively (P<0.001 for each). In the case of the less virulent AP53/CovR^+^S^+^, Manfredo M5/CovR^+^S^+^ and AP53/CovR^+^S^+^/ΔM53 strains, little dissemination was observed (data not shown), relative to the WT M1T1 5448 strain, but, nonetheless, bacteria were recovered from the skin to assess whether CovS switching could occur at the wound sites in these strains.

### CovS mutants were identified in GAS 5448/CovR^+^S^+^ infections

Strains with SpeB^−^ phenotypes are typically characterized by mutations in the *covR* or *covS* gene, with a preponderance of alterations in the latter gene [29], [30]. In order to determine whether the *in vivo* mutations in the *covS* gene reveal a pattern, several SpeB^−^ isolates were recovered from separately infected mice and their *covS* genes were sequenced. Samples were obtained not only from wound sites, but also from spleens, since we hypothesized that deeper tissue isolates would contain more virulent mutants. Plating tissue homogenates on THY-milk/agar plates allowed the ability to rapidly screen for SpeB activity by visually observing the switch from the lytic zone found around the SpeB^+^ colonies to the lack of such zones in SpeB^−^ colonies. Random colonies that appeared to be SpeB^−^ (no clearing) and β-hemolytic on blood agar (not shown), were further analyzed for SpeB proteolytic activity in concentrated culture supernatants using a spectrophotometric assay. Each infected mouse yielded several GAS clonal isolates, yet only a small percentage were SpeB^−^, with the remaining being SpeB^+^, similar to the injected sample (Table 2).

**Table 2:** Mutations found in *covS* gene of GAS strain 5448/*covS* ^+^ during infection in mice.^a^

Mouse	Tissue^b^	Gene Mutation	NT^c^	# bp^d^	# clones^e^	Protein Mutation^f^
1	Spleen	Deletion	974–1140	167	1	Delete 325–380
	Spleen	Deletion	1243	1	2	Stop-458
2	Spleen	Deletion	1243	1	2	Stop-458
3	Spleen	Deletion	279–297	19	10	Stop-156
4	Spleen	Deletion	279–297	19	3	Stop-156
	Spleen	Insertion	96	11	2	Stop-39
	Spleen	Mutation	404–405, 845	3	2	S135F, L282S
5	Spleen	Insertion	468	1	7	Stop-165
6	Spleen	Insertion	1357	1	7	Stop-478
	Spleen	Deletion	83	1	3	Stop-36
7	Spleen	Deletion	229–840	612	2	Delete 77-280
	Skin	Deletion	229–840	612	4	Delete 77-280
8	Spleen	Deletion	229–840	612	6	Delete 77-280
	Skin	Deletion	229–840	612	3	Delete 77-280
9	Spleen	Deletion	229–840	612	5	Delete 77-280
	Skin	Deletion	229–840	612	1	Delete 77-280
10	Spleen	Deletion	229–840	612	5	Delete 77-280
	Skin	Deletion	229–840	612	1	Delete 77-280
11	Spleen	Deletion	229–840	612	2	Delete 77-280
12	Spleen	Deletion	1104–1140	37	6	Stop-377
	Skin	Insertion	140	31	1	Stop-71
13	Spleen	Deletion	229-840	612	1	Delete 77-280
	Spleen	Insertion	160	31	1	Stop-71
	Skin	Insertion	998	4	1	Stop-339
14	Spleen	Insertion	160	31	1	Stop-71
	Spleen	Deletion	1104–1140	37	1	Stop-377
	Skin	Deletion	1211–1215	5	1	Stop-407
15	Spleen	Deletion	1104–1140	37	1	Stop-377
	Skin	Insertion	96	11	1	Stop-39
16	Skin	Insertion	140	31	2	Stop-71
17	Skin	Deletion	1376–1382	7	1	Stop-466

a Putative domains of CovS as defined elsewhere [8], [11], where mutations occur: CovS: TMI (22–33); EC Loop (34–180); TMII (181–205); HAMP (208–266); HisKA (269–333); HATPase (386–492).

b Tissue from which clones were obtained.

c Nucleotide (NT) position of change, or range of change, based on position of the *covS* start codon.

d Number of nucleotides of WT-*covS* replaced, inserted, or deleted.

e Number of clones sequenced.

f Effect on CovS protein.

The great majority of CovS^−^ mutations in recovered SpeB^−^ colonies were either deletions or insertions of 1 to 612 base pairs (bp) of DNA (Table 2). These changes occurred within the putative functional CovS protein domains (see footnote of Table 2 [8], [11]). The regions were established based on sequence comparison to similar histidine kinase sensor proteins and include two transmembrane regions (TMI and TMII), an extracellular sensor loop (EC Loop), a histidine kinases/adenylyl cyclases/methyl-binding proteins/phosphatases (HAMP) region, a histidine kinase or phosphoacceptor (HisKA) region, and a histidine kinase-like ATPase (HATPase) region [8], [11]. Of the 17 different mutations identified in spleen, and/or skin isolates, 6 occurred solely in the HATPase domain and a single isolate contained a mutation in the HisKA portion of the protein. The remaining isolates possessed mutations in multiple domains or in regions between the assigned domains. A total of 11 mutations resulted in premature truncation, either from in-frame or out-of-frame deletions or insertions, which led to the occurrence of premature translation stop codons. However, one of the mutations, identified from strains recovered from six different mice (#7-11 and #13), as well as a distinct mutation in another mouse (#1), represented large deletions within the gene that still terminated with the original stop codon. Only one of the mutants, 5448/#4, had two in-frame nucleotide point mutations. While many other mutations are likely to be present in the CovS^−^ strains that were not tested, our aim was to examine whether commonality existed in the types of mutants that were randomly isolated.

From these results, we conclude that there does not appear to be a particular motif or region of the *covS* gene that is not susceptible to mutation, but it is of interest that the same large DNA deletion occurred in multiple mice (#7–11 and #13), suggesting that this was a more susceptible mutagenic region of the gene. Six identical *covS* mutations appeared in GAS isolates from multiple mice. Strikingly, mutant strains #12 (skin) and #14 (spleen) have the same 31 bp insertion, which is located at different positions of the DNA, *viz.*, bp-140 and bp-160, respectively. Thus, the overall distribution of mutations encompasses most of the *covS* gene.

### CovS mutations were not identified in the GAS 5448/covR^+^S^+^ inoculum prior to infection

**Figure 2:**
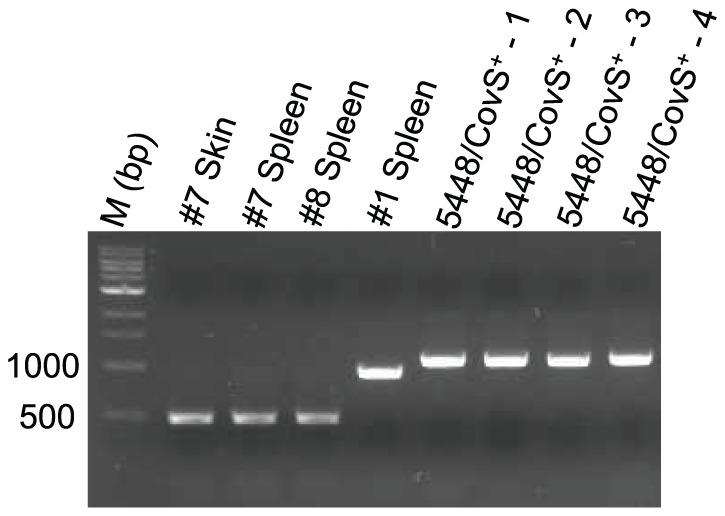
PCR of GAS 5448/covR^+^S^+^ inoculum prior to infection. Primers specific for regions of the *covS* gene that did not undergo mutations, but whose products would reveal the presence of a mutation, were used for PCR analysis. Genomic DNA isolated from overnight cultures of four different colonies of 5448/CovR^+^S^+^ was tested to determine if any mutations were present prior to injection. Mutant strains identified in this study were used as controls in lanes 2–5.

Since several covS mutations resulted in significantly smaller covS genes, *e.g.*, Mouse 1 Spleen (167 bp deletion) and Mouse 7 Spleen (612 bp deletion), the possibility that these mutations were present in the inoculum prior to infection was determined. A set of primers was designed such that the full-length covS gene would generate a ∼1102 bp band and the deletion mutants would generate bands corresponding to loss of 612 bp or 167 bp. Figure 2 shows the results of the extended PCR analysis on genomic DNA isolated from overnight cultures. It is clear that each lane contains a single PCR product that corresponds to the size of *covS* gene, suggesting that the initial inoculum contains bacteria with only full-length *covS* and that mutations arise and are selected for during the onset of infection.

### Identification of covS mutants in GAS AP53/CovR^+^S^+^ infections

**Figure 3:**
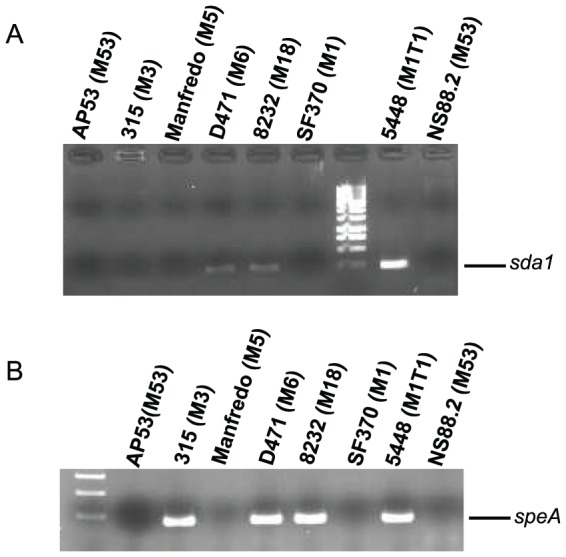
PCR of gDNA. Primers specific for the (A) *sda1* gene and (B) *speA* gene were used for PCR analysis of genomic DNA. The GAS strains employed are indicated in the figure along with the *emm* serotype for each of these strains (in parenthesis). M refers to the molecular size marker.

Since GAS 5448/CovR^+^S^+^ is highly virulent and has prophage genes lysogenically inserted, it is possible, and has indeed been proposed, that the acquisition of separate prophage regions encoding the streptodornase gene, *sda1*, and/or the superantigen *speA* gene, in synergy with the genetic scaffold of the clonal 5448/CovR^+^S^+^, stimulates CovS switching [31]. According to our DNA analysis of multiple strains of GAS, we find clear evidence for genomic full-length *sda1* in the *emm1* strain, 5448, along with other GAS strains with different M-protein serotypes, *e.g.*, *emm6* (D471) and *emm18* (MGAS8232) (Fig. 3A). We did not identify *sda1* in our clonal *emm3* strain (Fig. 3A), suggesting that this particular GAS strain had not acquired the *sda1*-containing prophage. Also, we found that the phage displayed a full-length *speA* gene in GAS *emm1* (5448), *emm3* (MGAS315), *emm6* (D471), and *emm18* (MGAS8232) (Fig. 3B). Other GAS strains, *e.g.*, *emm1* (SF370) and *emm5* (Manfredo), as well as the two closely related *emm53* serotype strains, AP53 and NS88.2, do not contain either of these particular prophage DNA inserts (Fig. 3B). The parent nonclonal *emm1* strain of 5448, *viz.*, SF370, also did not express *sda1* or *speA* (Fig. 3A,B). Thus, the genes, *sda1* and *speA*, originate from different phage insertions [32], of which 0, 1, or both can exist in other GAS strains, along with other types of prophage acquisitions.

We next sought to determine whether the virulence incorporated into the clonal GAS 5448 by the prophage encoded genes, and/or the particular M1 protein, was responsible for CovS^+^ switching. Both possibilities have been brought forward [33], [34]. Therefore, a variant of the human isolate, AP53/*covS^−^*, was employed which was generated by reinsertion of the deleted T^1404^ into this *covS* ^−^ to restore WT CovS^+^ and provide AP53/CovR^+^S^+^ [21], which is not virulent in the mouse model used [35], to assess whether *covS* switching can reoccur in a nonvirulent GAS strain. This variant not only possesses an intact *covS^+^* gene, but also does not contain the prophage-inserted genes, *sda1* and *speA* [35], and has a very different functional M-protein, *emm53*, than the M-protein, *emm1*, found in GAS 5448. In the case of AP53/CovS^+^ infections, we only examined GAS clones within the wound, using the same methodology described for study of 5448/CovR^+^S^+^. While, qualitatively, far fewer SpeB^−^ clones were observed *via* screening (∼5–10% of the total), five different CovS^−^ variants in five different mouse wounds, initiated through subcutaneous administration AP53/CovR^+^S^+^, were randomly identified. These mutants were positioned in a narrow range of the C-terminal translated sequence of CovS (Table 3), thus showing some mutagenic preference for this area of the gene, at least in this GAS strain. These mouse mutants are in addition to the original patient isolate, AP53/*covS* ^−^, which contains a sixth known *covS* inactivating mutation, specifically an in-frame deletion of T^1404^ in *covS*, generating a premature stop codon in *covS* at amino acid sequence position-469 [21]. Thus, CovS switching occurs with variable frequencies independent of the nature of the M-proteins and independent of the presence of the prophage genes, *sda1* and *speA*. While switching in these latter types of GAS may represent more rare events, it nonetheless explains the appearance of *covS^−^* genes, and the appearance of clonal virulent strains that have emerged from different types of nonvirulent strains of GAS. However, while the type of M-protein may not govern phase switching, a M-protein is required for CovS switching, since we found no SpeB^−^ isolates after several AP53/CovS^+^/ΔM53 infections.

**Table 3:** Mutations found in *covS* gene of GAS strain AP53/*covS^+^* during infection.

AP53 Clone	Tissue	#Mice or Humans with Mutation	Gene Mutation	NT^a^	# bp^b^	Protein Mutation^c^
1^d^	Mouse Skin	1	In-frame replacement	1205	12	Stop-406
2^d^	Mouse Skin	1	Frame-shift replacement (1 bp insert)	1205	20	Stop-410
3	Mouse Skin	1	Single bp replacement	1162	1	Stop-388
4	Mouse Skin	1	Frame-shift deletion	1432-1453	22	Stop-497
5	Mouse Skin	1	In-frame replacement	1399	1	Stop-467
6	Human Invasive Wound	1	Deletion	1404	1	Stop-469

a Nucleotide (NT) position of change based on position of *covS* start codon, ATG.

b Number of nucleotides of WT-*covS* replaced, inserted, or deleted.

c Effect on CovS protein.

d 1 and 2 were isolated from wounds in the same mouse.

### Identification of covS mutants in GAS Mandfredo/CovR^+^S^+^/emm5 infections

**Figure 4:**
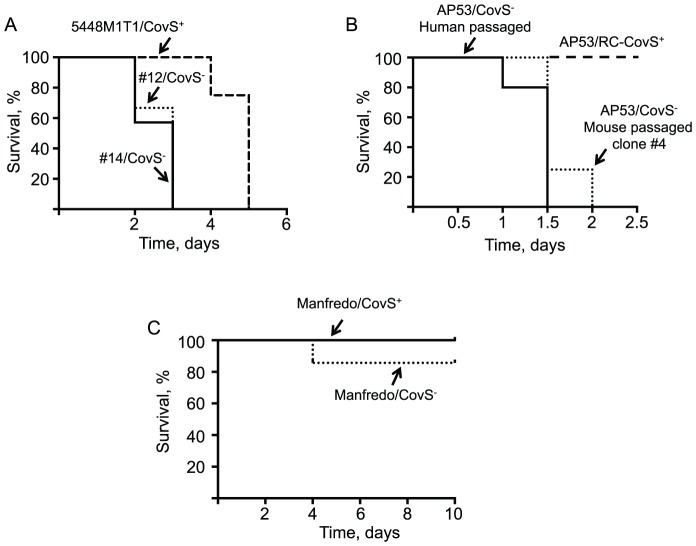
Survival of hPg-transgenic mice after infection with mouse-passaged GAS strains. C57Bl/6[hPg(Tg)] male mice (6–10 weeks of age) containing the hPg transgene were injected subcutaneously with GAS cells and the mice were monitored for death as an end-point. (A) 5448/CovR^+^S^+^/M1^+^, and mouse-passaged 5448/CovR^+^S^−^/M1^+^ strains #12 (skin) and #14 (skin). A total of 1.2×10^7^ cells were injected. P<0.05 when comparing the wild-type strain to the two MP strains. (B) AP53/CovR^+^S^−^/M53^+^ (human passaged) and AP53/CovR^+^S^−^/M53^+^ clonal strain #4 (Table 3) obtained after mouse passaging of AP53/CovR^+^S^+^/M53^+^. A dose of 1×10^8^ cells was injected. P<0.05 when comparing the covS^+^ strain to the human passaged or mouse passaged covS mutants. (C) Manfredo/CovR^+^S^+^/M5^+^ and Manfredo/CovR^+^S^−^/M5^+^ clonal strain #3 (Table 4) obtained after mouse passaging of Manfredo/CovR^+^S^+^/M5^+^. A total of 1.4×10^7^ cells were injected. These two curves were not statistically different. N = 4–8 mice for each tested strain.

The possibility of phase switching was also assessed in the more avirulent GAS Mandfredo/CovR^+^S^+^ strain that contains a fibrinogen binding M5 protein, and did not incorporate phage-encoded SpeA or Sda1 (Fig. 3). After infection, no lethality or deep tissue dissemination was observed. Thus, we screened GAS at the wound site and found a low percentage (<3%) of SpeB^−^ clones, which were randomly sequenced. The data show (Table 4) that switching to CovS^−^ strains did occur at the wound site. In one mouse, a SpeB^−^ clone (#1) with a truncation mutation was found near the amino-terminus. In a second mouse three SpeB^−^ clones were found with an in-frame deletion of Lys-263. Of interest, this single amino acid deletion abrogated SpeB production. In this same mouse, another SpeB^−^ clone was found with a five nucleotide deletion that was translated to a stop codon at amino acid 407. Comparison of survival of Manfredo/CovR^+^S^+^ with Manfredo CovR^+^S^−^ (clone #3, Table 4) did not show a significant difference (Fig. 4C), suggesting that in this low virulence strain, the inactivation of CovS is insufficient in itself to generate a hypervirulent strain of GAS.

**Table 4:** Mutations found in *covS* gene of GAS strain Manfredo M5/*covS^+^* during infection.

Mouse #	Tissue	Gene Mutation	NT^a^	# bp^b^	Protein Mutation^c^
1	Mouse Skin	Truncation	83	1	Stop-36
2	Mouse Skin	In-frame deletion	787–789	3	Delete K263
3	Mouse Skin	In-frame deletion	787–789	3	Delete K263
4	Mouse Skin	In-frame deletion	787–789	3	Delete K263
5	Mouse Skin	Deletion	1215–1219	5	Stop-407

a Nucleotide (NT) position of change based on position of *covS* start codon, ATG.

b Number of nucleotides of WT-*covS* replaced, inserted, or deleted.

c Effect on CovS protein.

### Mouse survival is diminished in covS^−^ switched strains of GAS

To prove that these CovS^−^ mutants were capable of high virulence, some of the isolated strains were examined for lethality in C57Bl/6[hPg(Tg)] mice. It has been shown that a WT *covS* gene (CovS^+^) indirectly represses expression of a number of virulence factors, including SK, HasABC, and SLO [36], [37], and ineffective CovS regulation of CovR leads to an increase in virulence and death [21]. In the case of GAS 5448/CovR^+^S^+^, the two mouse-passaged CovS^−^ mutant strains tested here showed a small, but clear (p<0.05), decrease in mouse survival relative to the 5448/CovS^+^ strain (Fig. 4A). This is not unexpected, as this strain contains the inserted prophage virulence enhancer genes, *speA* and *sdaI*, rendering 5448/CovR^+^S^+^ a highly virulent strain. Nonetheless, the lack of a WT *covS* ^+^ gene appears to provide an increased ability of GAS to infect and kill their host, with all 5448/CovR^+^S^+^ injected mice expiring within five days and all 5448/CovR^+^S^−^ injected mice, infected with clones #12 (skin) or #14 (skin), expiring within three days.

Since the 5448/CovR^+^S^+^ strain was already highly lethal prior to mouse passage, another GAS strain, AP53/CovR^+^S^+^, was examined, which does not show lethality in this model [35], and does not possess prophage-expressed Sda1 or SpeA, to assess the effects of MP CovS^−^ isolates on its virulence. The example MP GAS chosen for virulence studies was clone #4 of Table 3. The survival data show that clone #4 was as virulent as the human-passaged AP53/CovR^+^S^−^, with all mice expiring within 2 days (Fig. 4B). AP53/CovR^+^S^+^-infected mice remained viable in this model, as shown earlier. These data demonstrate that after infection, nonvirulent GAS can undergo switching, *via* transcriptional regulatory gene alterations, to provide hypervirulent strains, and that this mechanism is a critical evolutionary adaptation of GAS for survival in the human host.

### DNase activity and transcription are enhanced in phase switched GAS

**Figure 5:**
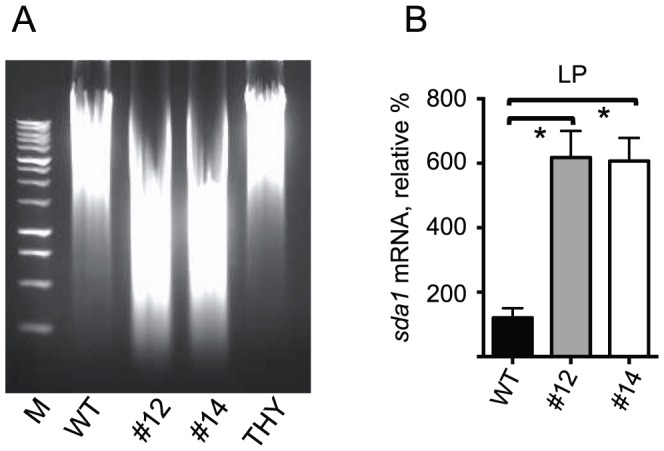
DNase activity and transcript levels in WT-5448/CovR^+^S^+^ and mouse-passaged clones of 5448/CovR^+^S^−^ in culture supernatants. (A) Cultures of WT-5448/CovR^+^S^+^ and clonal mouse-passaged 5448/CovR^+^S^−^ isolates #12 (skin) and #14 (skin) were grown to mid-log phase (LP; A_600nm_ ∼0.6) and their supernatants monitored for DNase activity. Supernatants were incubated with 1 µg of calf thymus DNA for 5 min at 37°C. DNase activity was confirmed by visualizing DNA fragmentation by ethidium bromide staining and UV-light detection. THY in lane 5 represents the negative control. *B*, The effect of mouse-passaged GAS 5448 on *sda1* gene expression is shown using mRNA isolated from washed cells harvested at mid-log phase (LP; A_600nm_ ∼0.6) and early stationary phase (SP; A_600nm_ ∼1.2) growth. WT-5448/CovR^+^S^+^ and mouse-passaged isolates (5448/CovR^+^S^−^) #12 (skin) and #14 (skin) were employed. The relative % gene expression levels of the mouse-passaged strains are relative to those in WT-5448/CovR^+^S^+^.

Extracellular DNase activity has been shown to be essential for degradation of neutrophil extracellular DNA traps (NETs), and CovS^−^ mutants of 5448 displayed an increased amount of DNase activity in their supernatants [34]. Upon examination of the DNase activity of the LP growth supernatants of MP clonal isolates #12 (skin) and #14 (skin), it is clear that calf thymus DNA was much more efficiently degraded in these strains than the supernatants from the WT CovR^+^S^+^ strain (Fig. 5A), the latter of which is near the negative control (THY media only). These results demonstrate that the increased DNase activity exhibited by CovS^−^ mutants of 5448 contribute significantly to this end-point of the host immune response.

To show that the increased DNase activity, shown in Figure 5A, of MP mutants #12 and #14, was consistent with increased DNase activity of the specific nuclease, Sda1, mRNA levels of the *sda1* gene were measured using RT-PCR. Total RNA was isolated from 5448/CovR^+^S^+^, and the two representative 5448/CovR^+^S^−^ MP mutants, #12 and #14, and assayed with primers specific for *sda1*, along with a reference gene, *gapdh* (*plr*). It is clear from Figure 5B that the expression ratios of *sda1* in the two mutant strains are much higher, by ∼6-fold, when compared to the level of *sda1* in 5448/CovR^+^S^+^ cultures. Increased expression of *sda1* correlates with increased enzymatic DNase activity observed in Figure 5A, and confirms the role of CovS in *sda1* gene regulation.

### CovS inactivating mutations diminish SpeB expression and enhance SK activity

**Figure 6:**
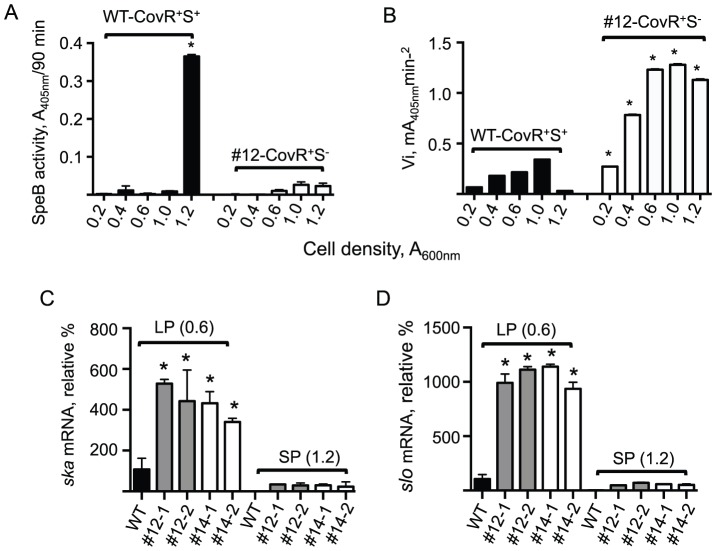
Gene expression in WT-5448/CovR^+^S^+^ and mouse-passaged clones of 5448/CovR^+^S^−^ in cells and in culture supernatants. (A) Levels of SpeB enzymatic activity measured spectrophotometrically in conditioned culture supernatants of WT-5448/CovR^+^S^+^ and mouse-passaged 5448-CovR^+^S^−^ strain #12 (skin) from Table 2. Aliquots of the supernatants were sampled at various cell densities, measured as A_600nm_. 5448/CovR^+^S^+^ shows the highest amount of SpeB activity when grown to early-SP (A_600nm_ = 1.2). Very little SpeB is measured during LP (A_600nm_ = 0.2–1.0). The data are expressed as A_405nm_, reflecting hydrolysis of the chromogenic substrate, after 90 min incubation. (B) Initial rates (V_i_) of hPg activation by culture supernatants sampled at various cell densities, reflecting SK activity in WT-5448/CovR^+^S^+^ and mouse-passaged 5448/CovR^+^S^−^ strain #12 (skin). Higher amounts of SK are present in conditioned culture media of 5448/CovR^+^S^−^ strain #12 than in that obtained from growth of WT-5448/CovR^+^S^+^. *P<0.01 in a two-tailed comparison of each measurement between WT-5448/CovR^+^S^+^ and 5448/CovR^+^S^−^ strain #12 at the same cell densities. (C-D) The effect of mouse-passaged GAS 5448 on (C) *ska* and (D) *slo* gene transcription. Total RNA was isolated from washed cells harvested at mid-LP (A_600nm_ ∼0.6) and early-SP (A_600nm_ ∼1.2) growth. WT-5448/CovR^+^S^+^ and 2 independent clones from mouse-passaged 5448/CovR^+^S^−^ #12 (#12-1 and #12-2) and #14 (#14-1 and #14-2) were employed for the RT-PCR assays. The % gene expression levels the mouse-passaged strains are relative to those in WT-5448/CovR^+^S^+^.

The levels of SpeB and SK activities were determined in both WT 5448/CovR^+^S^+^ and mouse-passaged strain #12 (5448/CovR^+^S^−^) at various stages of cell growth that spanned early log-phase (LP; A_600nm_ = 0.6) through stationary phase (SP; A_600nm_ = 1.2). Figure 6A shows that SpeB activity is only observed in the WT 5448 CovR^+^S^+^ strain grown to SP. SpeB is not observed to any great extent at any time during cell growth of the MP strain, 5448/CovR^+^S^−^ clone #12.

Figure 6B shows the amounts of SK activity present in the culture supernatants of the M1T1, determined as initial rates of hPg activation by conditioned GAS culture supernatants removed at various stages of growth. SK levels in supernatants of 5448/CovR^+^S^−^ clone #12 are higher at all growth stages than WT-5448/CovR^+^S^+^ in conditioned media and is primarily produced during log-phase (LP) growth. While *ska* mRNA levels increase ∼5-fold in two separate clones of MP strains 5448/CovR^+^S^−^ #12 and #14 in cells extracted at mid-LP, little detectable *ska* mRNA is present in 5448/CovR^+^S^−^ cell extracts at early-SP (Fig. 6C). Thus, little new SK expression occurs during SP growth of 5448/CovR^+^S^−^. The fact that SK protein levels are high at SP growth of 5448/CovR^+^S^−^ (Fig. 6B) is due to a combination of the accumulated SK during growth and the fact that SpeB, which is known to degrade active SK [38], is greatly reduced in 5448/CovR^+^S^−^ at all growth times. Thus, downregulation of SpeB in culture supernatants of 5448/CovR^+^S^−^, allows SK to persist during the SP in these latter supernatants.

To ascertain that control of other virulence genes is sustained during MP of the WT-5448 strain, and to provide credible evidence, to the extent practicable, that only *covS* is undergoing *in vivo* mutagenesis, the levels of an additional extracellular virulence-determinant, *viz.*, the toxin gene, *slo*, were determined in the MP strains. The *slo* gene encodes its product, streptolysin-O (SLO), an extracellular membrane pore-forming cytolysin that enhances another end-point of GAS virulence, *viz.*, the killing of host immune cells [39]. It is expected that this gene would be upregulated in GAS strains with enhanced virulence. As predicted, the data of Figure 6D show that the *slo* mRNA levels of two separate clones of MP GAS 5448/CovR^+^S^−^ #12 and 5448/CovR^+^S^−^ #14, grown to mid-LP (A_600nm_ ∼0.6), are highly upregulated, by ∼10 fold, as compared to WT-5448/CovR^+^S^+^ at the same growth stage. These data further show that *slo* mRNA is severely downregulated in each of these cell lines as growth proceeds to SP (A_600nm_ ∼1.2). At this latter point of growth, the mRNA levels of *slo* are severely depressed in both WT 5448/CovR^+^S^+^ and clones #12 and #14 of 5448/CovR^+^S^−^.

## Discussion and Conclusions

Many of the genes critical for GAS adherence, attachment, and invasion of its host are regulated by the CovRS system. In addition, this regulatory system affects genes involved in eliciting and evading immune responses [2], [35]. Hyperinvasive clinical isolates have been found to contain mutations in the *covS* gene, primarily causing a truncation in the protein with resulting loss of activity and, thus, regulatory function. Under non-stress conditions, CovR represses many virulence genes, and, with CovS inactivating mutants, CovR repression is shifted to a different set of targets, important among these is the extracellular cysteine protease, SpeB. SpeB has been associated with the degradation of a number of protein targets, originating from the host and from GAS, such as complement proteins, SK, and M-protein [38], [40], [41]. This combination of up-regulation of bacterial virulence, *e.g.*, assisting the bacteria with penetration of the host extracellular matrix, and reduction of SpeB mediated degradation of GAS virulence determinants increases persistence of many virulence-associated gene products and also suggests that this protease must be upregulated and downregulated at different times of infection in some strains. Clearly an additional advantage of SpeB expression would occur during an invasive phase since SpeB has been shown to degrade epithelial tight junction proteins [42]. Thus, selection for SpeB^−^, *via* CovS inactivating mutations, is a result of the bacterial evolution that assists its survival [33]. These types of mechanisms have allowed clonal variants of invasive GAS to develop and persist, accounting for the numerous hypervirulent strains that continue to be identified.

The majority of *covS* mutants thus far isolated have resulted in premature truncations in CovS that are contained in the HisKA or HATPase regionsat the C-terminal domains of CovS [8], [10], [33], [34]. Even if such truncated versions of CovS are expressed, this sensor would not have the ability to be phosphorylated, and thus could not regulate transcription *via* CovR. In addition, mutations in the *covR* gene have also been observed, the majority of which are point mutations that are believed to have no effects on DNA binding, but CovS phosphorylation is seemingly influenced [29], [43]. Differences have also been observed between full *covS* gene inactivations and animal-passaged generated *covS* mutants [44], suggesting that perhaps the remaining *covS* has retained some function, or the possibility that other gene changes take place during passage. Regardless, the goal of this work was to determine whether particular mutations in *covS* are favored after animal passage, and if a mechanism for such SpeB switching could be established.

In the case of hypervirulent M1T1 strain 5448/CovS^+^, mutations observed in our controlled study consisted primarily of either insertions or deletions of nucleotides. Deletions of single nucleotides or up to as many as 612 were observed. The insertions ranged from 1 to 31 nucleotides, and the larger insertions appear to be repeated sequences from the gene itself. For example, in mouse-passage strain #4, a clone is found with an 11 bp insertion at position 96, with the sequence GCATTTTCTCT. This sequence is a repeat of the full-length sequence immediately upstream. Previous studies have shown very similar mutations occurring in this same region, such as a reported 11 bp insert at position 103 from a CovS-deficient strain isolated from the blood of a patient with an infection [45]. A similar CovS mutant GAS strain was isolated from an animal-passage study that contained an 11 bp insert at position 99 [10]. In both cases, the inserts create repeated sequences that result in frame-shifts and premature truncation of the *covS* gene. GAS 5448 mutant clones #13 and #14 from this study contain a 31-nucleotide insertion, that, while not identical, are very similar to the sequence around the insertion location.

In addition to the large insertions, a number of mutants have been isolated with deleted regions of *covS*. The most common mutants represented in the literature involve deletion of 1–2 bp, although a number of others have been found with 5–10 bp deletions. In this work, mutants were recovered with deletions of >600 bp. This type of deletion allows for the possibility that during replication, strand separation and re-annealing of *covS* leads to some secondary structure formation resulting in misalignment. This type of slipped-strand mismatching is a common mechanism of phase variation in other bacterial species, and often arises in areas rich in simple sequence repeats (SSRs) or variable-nucleotide tandem repeats (VNTRs) [46]. Slipped-strand mismatching could explain both large and small insertions and deletions. It was recently shown that GAS strains that lack a 12 bp tandem repeat in the multiple gene regulator of GAS, *viz.*, the *mga* promoter region, were asymptomatic carrier strains, whereas strains with a 12 bp tandem repeat were much more virulent. This suggests a means to regulate the degree of infection via such a mechanism [47]. Here, we observe that 5448/*covS* mutants #12, #13, and #14 contain these VNTRs. In contrast to the *mga* inserts that result in modified regulation upstream of the coding sequence, *covS* coding region insertions and deletions result in truncation of the transcript and a non-functional protein, thus allowing for another type of phase variation mechanism in GAS.

It has been shown that SpeB switching can occur in a number of GAS strains and appears to occur independently of the M-protein-type [9]. Our studies are in accord with this and show that 5448/CovR^+^S^+^/M1, AP53/CovR^+^S^+^/M53, as well as Manfredo/M5, three diverse M proteins, generate CovS^−^ mutants. Also, neither AP53/CovR^+^S^+^ nor Manfredo M5/CovR^+^S^+^ contain the prophage lysogenic inserts that express Sda1 or SpeA, two widely accepted virulence factors that have been implicated in CovS^+^ phase switching. Since CovS switching occurs in AP53/CovR^+^S^+^ and Manfredo M5/CovR^+^S^+^, albeit at much lower rates than 5448/CovR^+^S^+^, it is obvious that these particular prophage genes are not absolute requirements for phase switching, although these genes may facilitate such events by being more inherently virulent and evading host defenses more effectively, thus exposing the bacteria to different compartments of the host and longer survival times in infected tissue. Also, considering the fact that AP53/CovR^+^S^−^ displays an invasive phenotype in a human CovS^−^ isolate, and in mice, it is clear that the virulent strain of AP53 is derived from the nonvirulent parent strain through CovS inactivating mutations. This conclusion is consistent with that of a previous study [7]. However, when the skin was infected with AP53/CovR^+^S^+^/ΔPAM, no evidence for SpeB^−^ clones in the skin wounds was found. Thus, M-protein, directly or indirectly, is necessary for this switching, but its exact nature may be less important.

Of the CovS^+^ strains examined, the M1T1 strain has a high propensity to phase switch from CovS^+^ to CovS^−^. While it was initially thought that the *sda1* gene, present in virulent *emm1* M1T1 strains, was critical for this switching mechanism to occur [8], insertion of the same gene into another non-virulent *emm1* strain, SF370, did not enhance its ability to switch to SpeB^−^, at least not at a high rate [31]. In addition to *sda1* and M-protein (*emm*), hyaluronic acid synthesis genes (*hasABC*) are believed to be important for the SpeB-switch to take place [34], likely by a more encapsulated GAS subverting host killing. Overall, the mechanism of how such genes enable the switching to occur more readily is unclear, although the ability to evade neutrophil phagocytosis and the host immune system seem to be critical. It is also unclear as to the properties of the M1T1 that confer a higher propensity to switch compared to other GAS strains, other than a possible role for enhanced virulence in a CovS^+^ strain.

In the MP CovS^−^ mutants, it was also determined whether other virulence genes were susceptible to mutation. Although a genome-wide analysis of GAS indicated that a 7 bp frameshift in CovS was sufficient to enhance survival during phagocytosis and to evade killing by polymorphonuclear cells [7] nonetheless, we assessed CovRS-responsive genes, *sda1*, *ska*, and *slo*, and indirectly *emm* and *hasABC*. None of these important GAS virulence genes underwent mutagenesis during MP, as determined by RT-PCR and/or protein analyses, and their expressions were controlled by CovRS. While, of course other genes that were not examined could undergo mutagenesis, it was felt that a good sampling was obtained by these choices of genes. However, since highly virulent GAS is either a laboratory-engineered strain generated from less virulent animal isolates, which are usually CovR^+^S^+^, or obtained as highly virulent isolates, which are usually CovR^+^S^−^, or are derived from isolates that contain virulent prophage inserts, *e.g.*, Sda1^+^/SpeA^+^, many gene changes have occurred in the animal host that are highly varied. Despite this, one general change that does occur in animal passage is CovS^+^ switching to many types of CovS^−^, and mechanisms that illuminate this point are of particular value.

While the AP53 strain showed a very large effect of CovS^−^ gene switching on virulence, as defined by death, the Manfredo strain did not show such an effect. This latter low virulence strain did not gain in this property as a result of CovS switching. There are many stages involved between infection and death that could require gene interactions between the host and microbe, and which may depend upon the total genetic architecture of the particular GAS strain. This fertile area requires study to reveal the mechanisms involved in overall virulence of the GAS.

In conclusion, the *covRS* operon appears to be a particular mutagenic target within the bacterial genome, and all regions of the *covS* gene are susceptible to mutations that inactivate this sensor component of this operon and thus assist GAS virulence. In addition to *covRS*, other such genomic mutagenesis targets exist in regulatory TCS genes [48], *e.g.*, *fasBCAX* and *ihk/irr*, and one-component gene regulators, *e.g.*, *rgg* [49], *ralp* [50], and *mga* [51]. While exceptions exist, overall, it is generally agreed that mutations found in such regulatory systems result in hypervirulent strains of GAS, and that these mutations arise under the selective pressures of survival in a host. The ability of GAS and other Gram+ pathogens to undergo selection *in vivo* leading to altered phenotypes is of high clinical significance. Bacteria are able to colonize human hosts for long periods of time. Thus, a greater understanding of the triggers and mechanisms that allow them to become more virulent is of significance to global health and to vaccine development.
